# Functional expression of the transient receptor potential ankyrin type 1 channel in pancreatic adenocarcinoma cells

**DOI:** 10.1038/s41598-021-81250-3

**Published:** 2021-01-21

**Authors:** Florentina Cojocaru, Tudor Şelescu, Dan Domocoş, Luminiţa Măruţescu, Gabriela Chiritoiu, Nicoleta-Raluca Chelaru, Simona Dima, Dan Mihăilescu, Alexandru Babes, Dana Cucu

**Affiliations:** 1 Department DAFAB, Faculty of Biology, University of Bucharest, Splaiul Independenței 91-95, Bucharest, Romania; 2 Faculty of Biology, Research Institute of the University of Bucharest (ICUB), University of Bucharest, Bucharest, Romania; 3 Department of Molecular Cell Biology, Institute of Biochemistry, Romanian Academy, Splaiul Independenței 296, 060031 Bucharest, Romania; 4 Center of Excellence in Translational Medicine, Fundeni Clinical Institute, 022328 Bucharest, Romania

**Keywords:** Collective cell migration, Ion transport

## Abstract

The transient receptor potential ankyrin type 1 (TRPA1) channel belongs to the TRP superfamily of ion channels. TRPA1 is a membrane protein with multiple functions able to respond to noxious stimuli, reactive oxygen species, inflammatory cytokines or pungent substances, and it participates in pain signalling, taste, inflammation and various steps of the tumorigenic process. To date, no reports have addressed the expression and function of TRPA1 in pancreatic ductal adenocarcinoma (PDAC) cells. This work reports the endogenous expression of TRPA1 channels in human pancreatic adenocarcinoma cell lines and provides insights into the function of the TRPA1 protein in the Panc-1 cell line. This study reports that cell lines isolated from PDAC patients had different levels of TRPA1 expression. The channel activity in Panc-1 cells, as assessed with electrophysiological (whole-cell patch clamp) and microfluorimetry methods, showed that non-selective cationic currents were activated by allyl isothiocyanate (AITC) in Panc-1 cells and inhibited by the selective TRPA1 antagonist A-967079. The current elicited by the specific agonist was associated with a robust increase in intracellular Ca^2+^. Furthermore, siRNA-induced downregulation of TRPA1 enhanced cell migration in the wound healing assay, indicating a possible role of ion channels independent from pore function. Finally, TRPA1 activation changed the cell cycle progression. Taken together, these results support the idea of channel-dependent and independent role for TRPA1 in tumoral processes.

## Introduction

Pancreatic ductal adenocarcinoma (PDAC) is one of the most lethal forms of cancer, and despite many efforts to address this dreadful disease, its 5-year survival rate is still approximately 9%^1^. The most evident reasons for such a poor prognosis are late diagnosis associated with asymptomatic patients and therapeutic resistance. Thus, a number of studies are investigating biomarkers for the early onset of the disease or new targets for the best treatment options. Among the many target proteins considered for exploitation with novel therapies are ion channels, which have recently been proposed as appealing druggable markers of the tumorigenic process^2^. This new avenue of oncological application is based on studies describing channels as transport proteins as well as important actors with pore-independent functions in signalling processes. For instance, the acid-sensing ion channels ASIC1 and ASIC3 mediate acidity-associated physiological and pathological events in PDAC tumours^3^ while high levels of Ca^2+^-activated chloride channels support PDAC cell migration^4^. Other studies have provided experimental evidence that members of the TRP channel superfamily are key factors in PDAC aggressiveness, prognosis and cancer cell invasion^5–7^. TRP channels not only participate in the uptake of Ca^2+^ and other cations but also interact with proteins involved in signalling pathways; thus, they are interesting players to study in carcinogenesis research. In addition, the ongoing development of agonists and antagonists of these channels paves the way for more precise pharmacological experiments, which will improve the accuracy of identifying these proteins when used as druggable targets.

The TRP superfamily consists of 6 subfamilies in humans. Transient receptor potential ankyrin type 1 (TRPA1), the only member of the ankyrin subfamily, has high Ca^2+^ permeability and is activated by various stimuli, such as cold temperatures, pungent compounds, reactive oxygen species and endogenous compounds^8^. This channel is overexpressed in some cancer types but has largely been overlooked by previous studies. An analysis of data from The Cancer Genome Atlas project shows that high expression of the TRPA1 gene correlates with improved survival in liver, intrahepatic bile duct and bladder cancers. However, the limited number of cases impedes a clear analysis of the putative role of TRPA1 as a diagnostic marker. Recently published results show that in breast and lung cancers, this channel protects tumour cells by increasing oxidative stress tolerance. However, little is known about the expression of TRPA1 in PDAC cells^9^.

The main aim of the present work was to determine the expression of TRPA1 in PDAC cell lines and to investigate whether this channel is functional in these cells. Moreover, we focused on the conducting roles of TRPA1 in the migration and cell cycle of PDAC cells as well as on the putative endogenous expression and activation of these channels. The results of this new study on TRPA1 expression in pancreatic adenocarcinoma cell lines provide insights into the function of TRPA1 channels in cancer and their putative role as participants to the cancerogenic process via channel-independent mechanisms.

## Results

**Figure 1:**
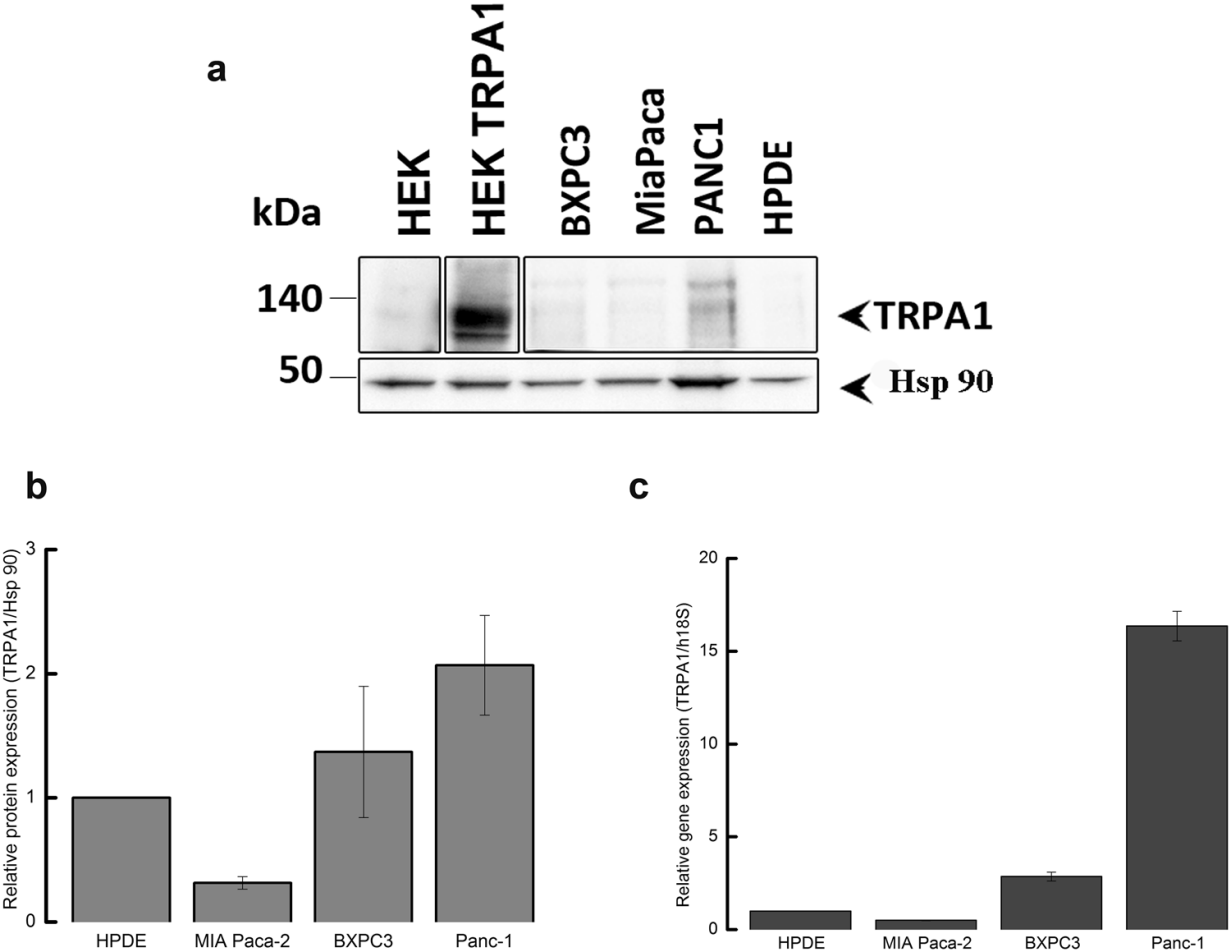
Expression of TRPA1 in human PDAC cell lines. (**A**) Expression pattern of the TRPA1 protein in PDAC cell lines. Blots from whole-cell lysates (50 µg) obtained from three PDAC cell lines, the human pancreatic ductal cells, wild-type HEK293T and HEK293T/A1-transfected cell lines were probed with antibodies against TRPA1 and Hsp90 on the same gel. Cropped gel is presented here with full gel available in Supplemental Fig. 1. (**B**) Densitometry quantification of the bands from panel A was performed with Quantity One software. The results are expressed as the ratio of the expression of TRPA1 to Hsp 90. (**C**) Levels of TRPA1 mRNA were determined by real-time RT-PCR, and a comparative Ct method (2^−ΔΔCt^) was used for the relative mRNA quantification. Hu18S was used as endogenous gene. The values in (**B**,**C**) are shown as the means ± SD; *n* = 3. ^∗^*p* < 0.05 vs. control.

**Figure 2:**
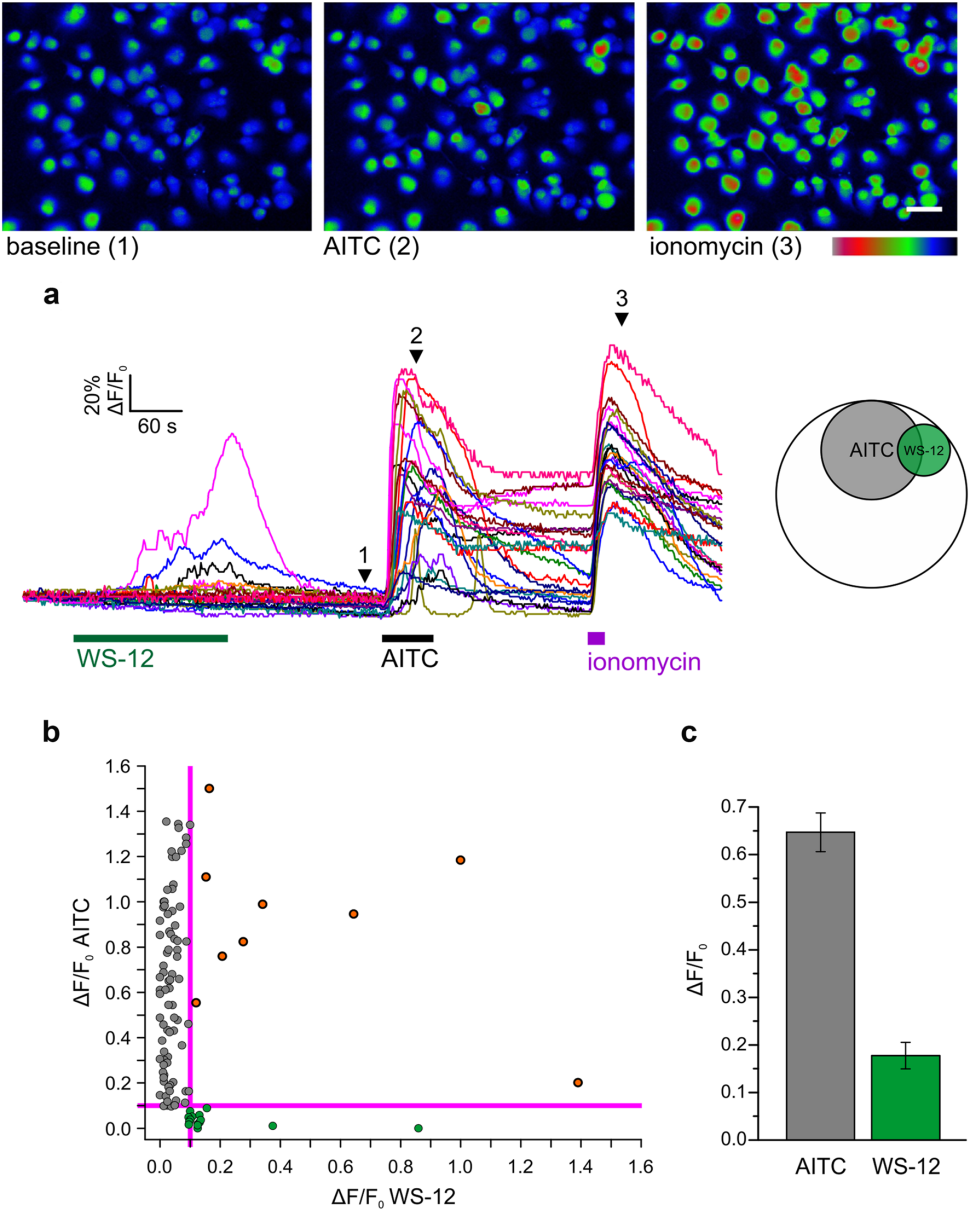
Calcium microfluorimetry shows functional TRPA1 and TRPM8 expression in populations of Panc-1 cells. (**A**) False colour fluorescence images extracted from a calcium microfluorimetry sequence at baseline during AITC stimulation (100 μM) and during treatment with ionomycin (2 μM) as a control at the end of the time course show that intracellular Ca^2+^ increased in a population of Panc-1 cells during stimulation with AITC. The white scale bar indicates 50 μm. Lower left panel: representative individual fluorescence traces from the same experiment during exposure to WS-12 (5 μM), AITC and ionomycin. The arrowheads and numbers indicate the acquisition times of the three images shown above. Lower right panel: Venn diagram showing the relation between AITC-responding cells (grey, 27%,) and WS-12-responding cells (green, 7.6%), which are surrounded by a large proportion of cells insensitive to both agonists (white, 67.5%, n = 329 cells examined). (**B**) Scatter plot displaying the relationship between response amplitudes to AITC and WS-12. Same cells as above (82 cells sensitive to AITC-only, 15 to WS-12 and 10 cells to both agonists). The threshold values for identifying a response were set at 10% ΔF/F_0_. (**C**) The average ± SEM of the response amplitudes to AITC and WS-12 (for 92 AITC-sensitive cells and 25 WS-12-sensitive cells; same cells as in (**A**,**B**)).

**Figure 3:**
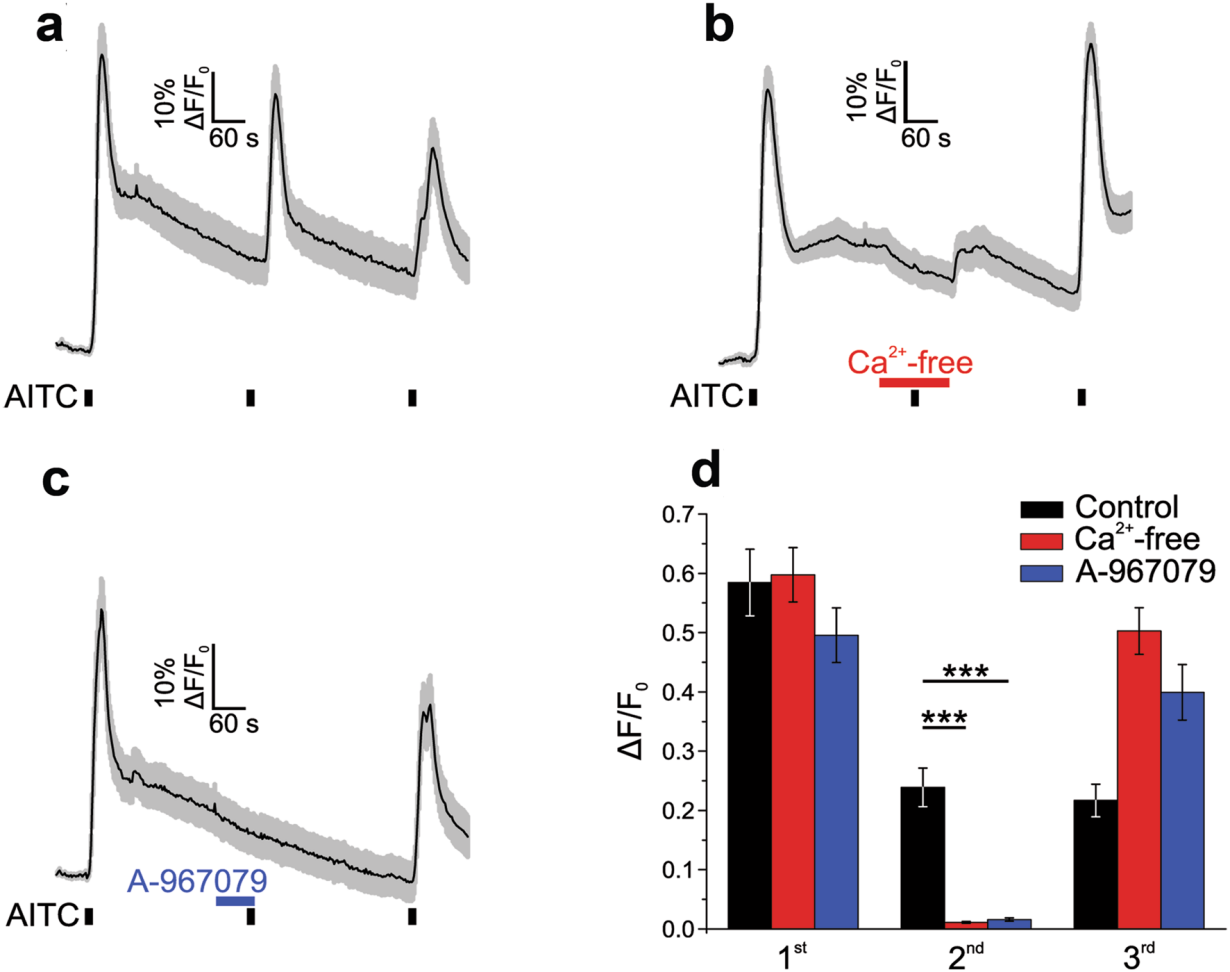
Calcium microfluorimetry shows functional TRPA1 and TRPM8 expression in populations of Panc-1 cells. (**A**) Average ΔF/F_0_ trace ± SEM (n = 21 cells) during control recordings across three AITC (25 μM, 15 s) challenges. (**B**) Replacement with a Ca^2+^-free extracellular solution before and during the 2nd AITC application abolished the response (average ΔF/F_0_ trace ± SEM from n = 65 cells). (**C**) The specific TRPA1 inhibitor A-967079 (10 μM) was applied before and then co-applied with AITC during the 2nd challenge and completely inhibited the response (average ΔF/F_0_ trace ± SEM from n = 31 cells). (**D**) Pooled data from the above experiments and statistical analysis. The average ± SEM ΔF/F_0_ values are shown for each AITC stimulus in the series (***p < 0.001, two-sample Student’s t-test; control, n = 45; Ca^2+^-free, n = 65; A-967079, n = 50).

**Figure 4:**
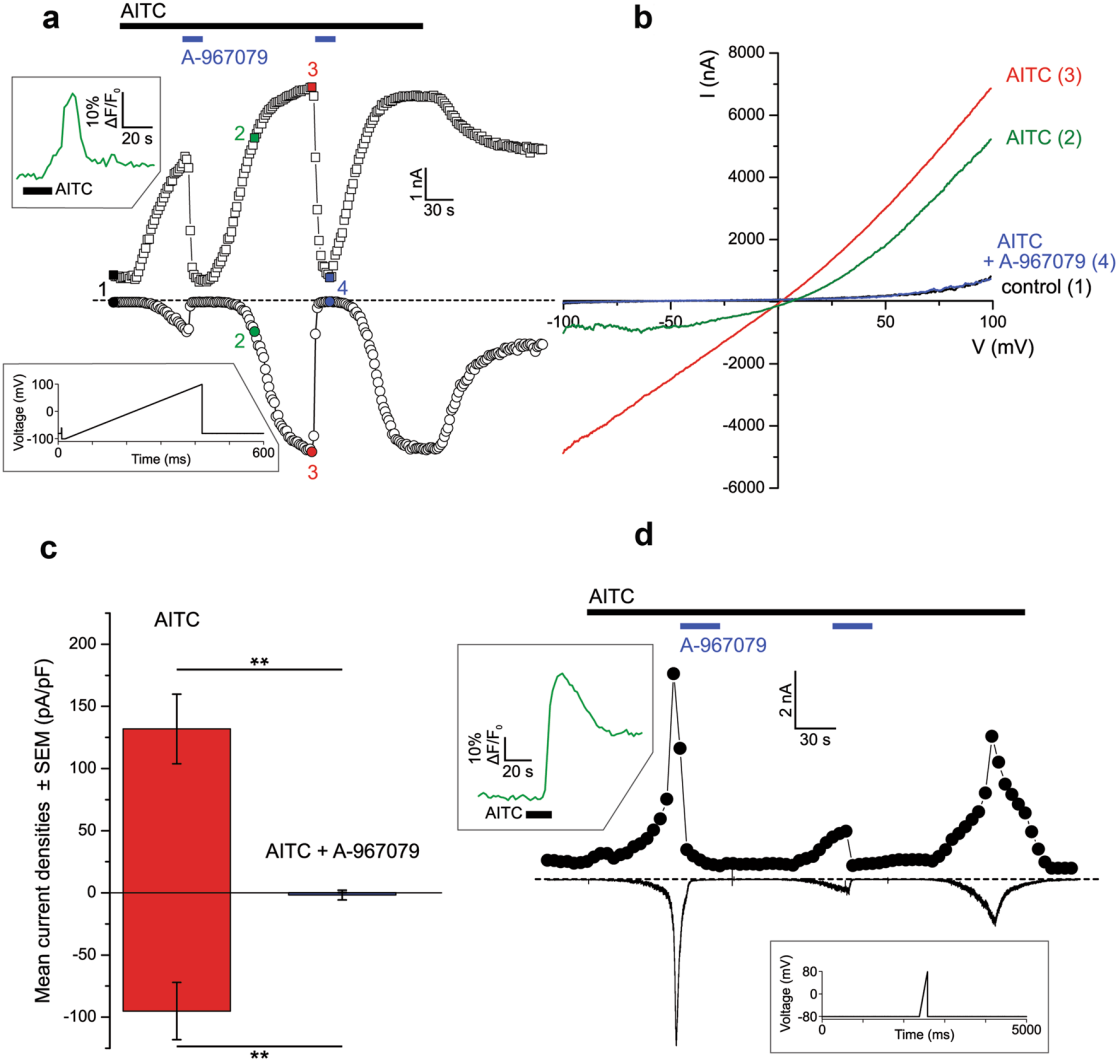
Whole-cell current recordings in Panc-1 cells confirm the functional expression of TRPA1 in this cell line. (**A**) AITC (25 μM)-sensitive cells were identified using calcium microfluorimetry (upper green trace from upper left inset). The solution was then replaced with the Ca^2+-^free variant, and the responding cells were patch-clamped. Representative example recording showing the time course of the peak outward and inward currents during superfusion with AITC (100 μM). During co-application of A-967079 (10 µM), the currents were almost completely eliminated. The voltage clamp protocol (ramping from − 100 to 100 mV) is shown in the lower left inset. (**B**) Current–voltage plots at the points indicated, with colours and numbers as shown in (**A**). Outward rectification regime is observable at sub-maximal current activation (trace 2), while the current at maximal activation (trace 3) shows an almost linear I-V relationship. (**C**). Pooled data from n = 6 recordings using the above protocols. Average current density (measured from baseline) ± SEM. A-966790 produced a statistically significant inhibitory effect (**p < 0.001, paired-sample Student’s t-test). (**D**) Representative recording performed in Ca^2+^-containing solution showing the time course of the peak outward current and inward current recorded at a constant holding potential of − 80 mV. The currents displayed a transient behaviour in response to AITC (25 μM). A-967079 (10 µM) inhibition is especially noticeable after the second current increase. The calcium microfluorimetry trace used for selecting the cell is displayed in the upper left inset, while the voltage clamp protocol (holding at − 80 mV and ramping from − 80 to 80 mV) is shown in the lower right inset.

**Figure 5:**
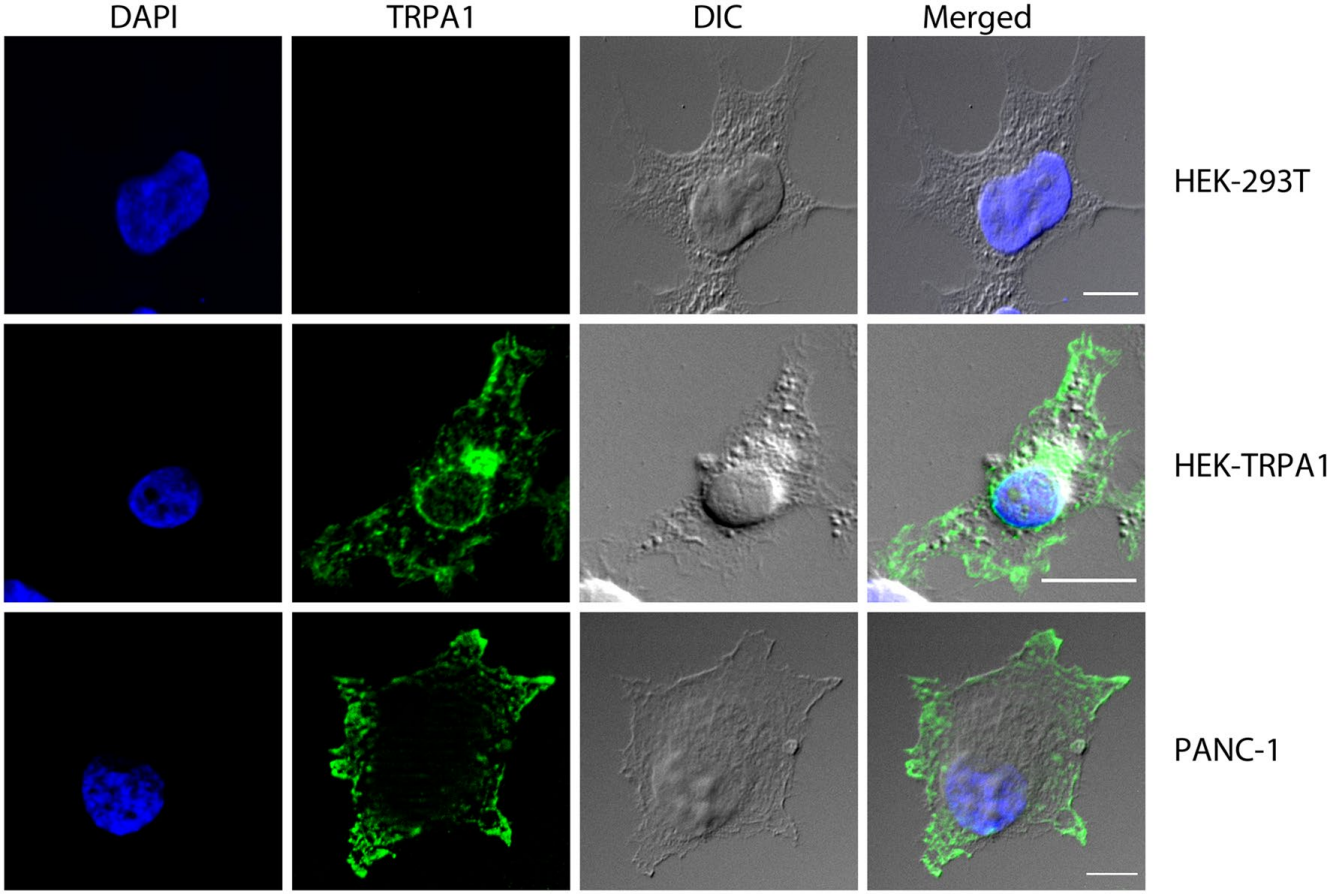
Expression of TRPA1 protein in PDAC cells A. Immunofluorescence of fixed Panc-1 and HEK-293 T cells either transfected or un-transfected with pcDNA3.1 plasmid encoding human TRPA1. Cells were stained with mouse monoclonal TRPA1 primary antibody (green), whereas DAPI nuclear staining is shown in blue. Scales are included on each image. Bars represent 10 µm.

- ***Expression of TRPA1 channels in PDAC cell lines***
  Previous studies reported that TRPA1 is detectable in glioblastoma, breast cancer and other various cancer types^9,10^. There are no reports on TRPA1 channel in PDAC, but the analysis of the Cancer Genome Atlas database reveals that TRPA1 is expressed in several patients with pancreatic cancer. However, data are not available on the expression of TRPA1 in human pancreatic cell lines.
  Using Western blotting (WB), we found that TRPA1 is expressed in the PDAC cell lines Panc-1, MIA Paca-2 and BxPC-3 (Fig. 1). Human embryonic kidney 293 T (HEK-293 T) cells transiently transfected with human TRPA1 were used as a positive control, whereas HEK-293 T wild-type cells were used as negative controls (Fig. 1A). Quantification of TRPA1 expression in PDAC cells was carried out in comparison to human pancreatic duct epithelial cell line (HPDE). The Western blot analysis revealed a specific molecular band with estimated size of about 130 kDa visible in BxPC3 and Panc-1 cell lines, comparable to those from transfected HEK-293 T cells. Lower expression levels were estimated in MIA Paca-2 and HDPE cells (Fig. 1A). Consistent with these results, relative low mRNA levels were detected in HPDE and MIA Paca-2 cells. However, TRPA1 mRNA levels were significantly higher in Panc-1 than in non-tumoral cells (Fig. 1B).
  Because the Panc-1 cell line is a good model for TRP channel expression^7,11^ and it has a robust TRPA1 expression we used it for further investigations. First, we aimed to investigate whether the channel is functional in Panc-1 cells.
- ***TRPA1 channel is functional in PDA cell lines***
  As previously reported^11^, Panc-1 cells express the TRPM8 protein. To determine whether this is true for the present batch of cells, we first applied the selective TRPM8 agonist WS-12 (5 μM for 3 min), followed by the TRPA1 agonist allyl isothiocyanate (AITC, 100 μM for 1 min, Fig. 2A). We confirmed that a population of cells (7.6% from n = 329 total cells examined) responded to WS-12, thus demonstrating functional expression of TRPM8 (Fig. 2A right). Moreover, AITC evoked robust Ca^2+^ transients in an even larger cell population (27% from n = 329 total cells examined), indicating that TRPA1 is also functionally expressed in Panc-1 cells (Fig. 2A right). The relationship between the sensibilities to WS-12 and AITC is shown in Fig. 2B_._ There was no significant dependency relationship between the responses to the two agonists, as evidenced by Fisher's exact test (*p* < 0.05, n = 222 cells insensitive to both agonists, 15 sensitive to WS-12 only, 82 to AITC only, and 10 to both agonists). The average ΔF/F_0_ amplitude of the responses to AITC (64.7 ± 4.1%, n = 92) was larger than the equivalent value for WS-12 (17.7 ± 2.8%, n = 25, Fig. 2C).
  To investigate the action of a selective TRPA1 antagonist and the contribution to extracellular calcium on the responses to AITC, we then employed a sequence of three AITC stimuli (15 s each at 5-min intervals), applied at a lower concentration (25 μM AITC), to exclude large desensitization effects. The control experiment is displayed in Fig. 3A. To confirm that the AITC-evoked Ca^2+^ transients were due to Ca^2+^ influx, the standard extracellular solution (ES) was replaced with a Ca^2+^-free solution during the 2nd AITC challenge (Fig. 3B). This replacement inhibited the response to the 2nd challenge by approximately 99% compared to the control (ΔF/F_0_ from 23.9 ± 3.3% in control, n = 45, to 1.1 ± 0.2 in Ca^2+^-free conditions, n = 65, *p* < 0.001, two-sample Student’s t-test; Fig. 3D). To confirm that the Ca^2+^ influx was mediated by TRPA1, the highly specific and potent TRPA1 antagonist A-967079 (10 μM) was applied during the 2nd AITC challenge (Fig. 3C), which inhibited the 2nd response by approximately 99% compared to the control response (ΔF/F_0_ from 23.9 ± 3.3% in control, n = 45, to 1.6 ± 0.3, n = 50, *p* < 0.001, two-sample Student’s t-test; Fig. 3D).
  To further examine the effect of TRPA1 activation, we recorded AITC-elicited currents in Panc-1 cells. First, we selected cells that showed robust responses of intracellular Ca^2+^ elevation upon stimulation with AITC (25 μM). The intracellular solution was based on potassium gluconate and used to isolate the TRPA1 current from currents through Cl^-^ channels. Under extracellular Ca^2+^-free conditions, AITC (100 μM) elicited sustained currents showing outward rectification (Fig. 4A, B) with a reversal potential of almost 0 mV (1.5 ± 2.3 mV, n = 6). At − 100 mV, the average density of the AITC-evoked current measured between − 163.1 and − 7.7 pA/pF, with a mean of − 95.2 ± 23.0 pA/pF (n = 6). The AITC-elicited current was repeatedly and reversibly inhibited by A-967079 (10 μM; Fig. 4A, B). Most likely, A-967079 washout and the AITC solution flow rate limited the speed of current recovery following the inhibition. During the second exposure to A-967079, the inward current density was inhibited by approximately 99% (from − 95.2 ± 23.0 pA/pF to 0.1 ± 0.2 pA/pF; *p* < 0.001, paired-sample Student’s t-test, n = 6), while the outward current density was inhibited by approximately 101% (from 131.8 ± 28.1 pA/pF to − 1.8 ± 4.0 pA/pF; *p* < 0.001, paired-sample Student’s t-test; n = 6; Fig. 4C). Inhibition greater than 100% suggests that TRPA1 had basal activity in Panc-1 cells before stimulation of the current with AITC. Under more representative physiological conditions with 2 mM external CaCl_2_, AITC (25 μM) evoked transient currents with fast acute desensitization, which decreased the suitability of the recordings for pharmacological assays. The inhibition mediated by A-96779 (10 μM) was more clearly observable when the inhibitor was delivered during the initially slower phase of current increase as illustrated in Fig. 4D during the second antagonist exposure.
  From these data we may assume that Panc-1 cells have low expression levels of TRPA1, although the currents evoked by TRPA1 agonist are quite robust. To clarify these results, we used confocal microscopy and immunohistochemical methods. Using an anti-TRPA1 human antibody, we identified TRPA1 in Panc-1 and HEK-A1, while un-transfected HEK293T cells are negative controls (Fig. 5A). Most of Panc-1 cells (over 80%) exhibited membrane associated patterns of TRPA1 when compared to control and transfected cells. Moreover, cells expressing the TRPA1 proteins show a strong expression similar to HEK-transfected cells.
  With these results we assume that there is a pool of TRPA1 channels that are sensitive to an endogenous activator factor, as previously shown in other systems^12^. We tested this hypothesis using cells kept in the A-967079 and performed calcium microfluorimetry while washing the inhibitor (Supplemental Fig. 2). Signals recorded in these conditions may be related to constitutively active channels. We obtained responses only in few cells (~ 10%), which are again too few to entirely support this hypothesis. Alternatively, it is possible that the endogenous activator may be washed out during perfusion of the extracellular solution.
  Another possibility is that TRPA1 in Panc-1 cells participate to tumoral processes in a pore-independent manner as previously shown for many channels, including TRPM8 and TRPM7^12–14^. Therefore, to investigate the role of TRPA1 independent of ion-conduction state, we performed experiments on migration using TRPA1-siRNA transfected and un-transfected cells.
- ***TRPA1 involvement in migration and cell cycle progression***

Previous reports indicating that ion channels contribute to cancer pathogenesis show that they may participate in the migration or proliferation of tumour cells independent of their conduction state through pathways that are incompletely understood^15^.

**Figure 6:**
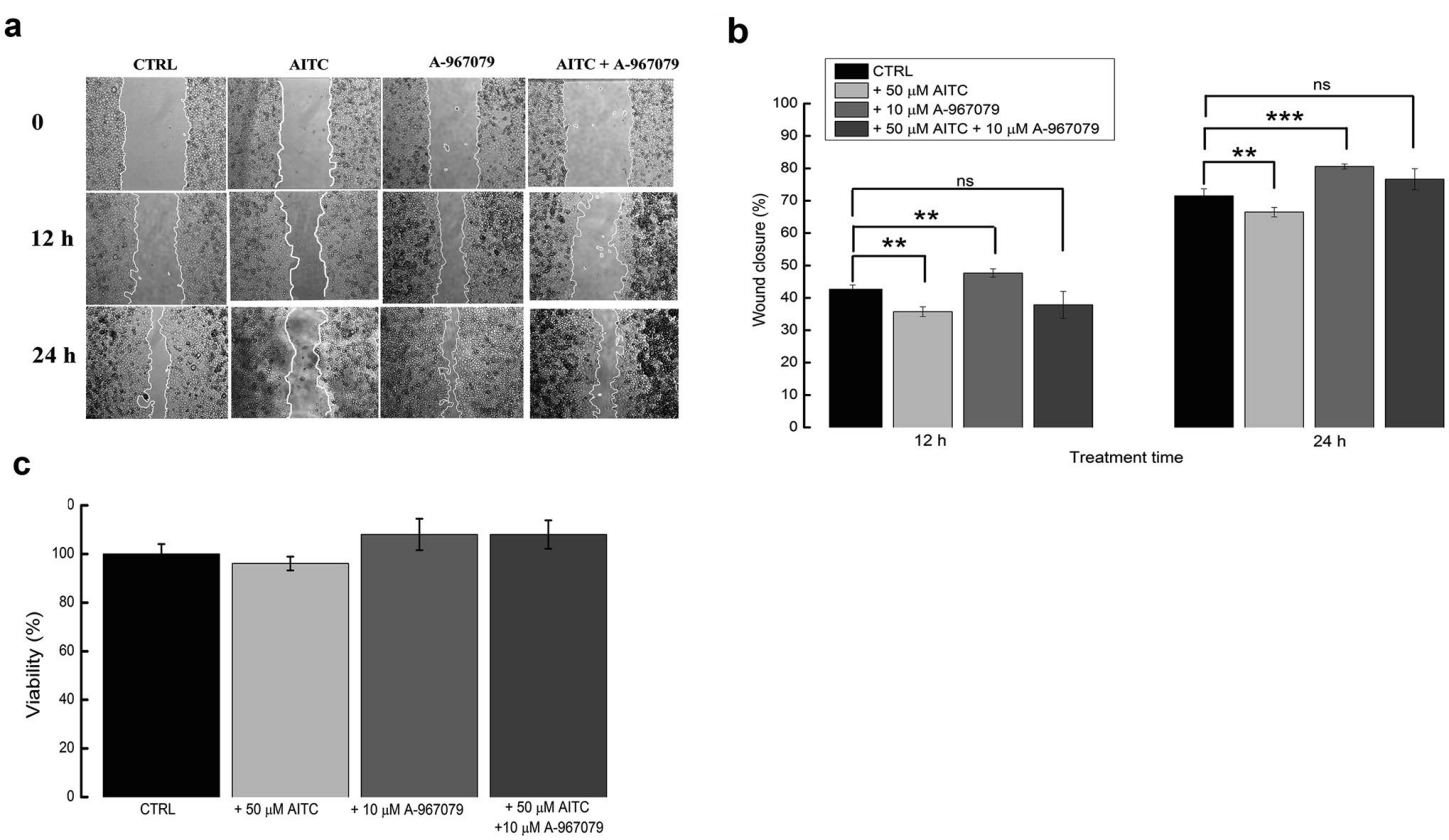
Effects of TRPA1 activation on tumour cell migration. (**A**) Wound healing assay. Panc-1 cells were grown to confluence in three Petri dishes per condition. The monolayer was wounded and imaged immediately (0 h). Growth medium containing 10 μM AITC, A-967079 (10 µM) or a mixture of both were added. Wound closure was recorded at 12 h and 24 h after scratch. The area of the wound was determined by carefully selecting the cell-free regions as displayed with white contours using ImageJ software. Average ± SEM values were calculated from 20 measurements per time point, and the wound healing percent was calculated at each time point (see also Method section). The experiment was repeated at least three times, and images from a representative experiment are shown. (**B**) Average ± SEM values from the three experiments presented in (**A**). ns, not statistically significant; *p < 0.05; **p < 0.01; ***p < 0.001 as calculated with Student’s t-test. (**C**) Cell viability after 24 h treatment with the indicated compounds. Bars represent 3 experiments, which were each performed in triplicate.

To determine whether TRPA1 contributes to cell migration when activated by its specific agonist, we performed wound healing assays with Panc-1 cells treated or not treated with AITC (50 μM). Moreover, to examine whether this activation could be abolished we used co-application of AITC with A-967079 (10 μM) (Fig. 6A). As shown in Fig. 6B, the TRPA1 agonist significantly inhibited cell migration from 42.66 ± 1.32% under control conditions to 35.73 ± 1.50% after AITC addition for 12 h (n = 6 experiments, Student’s unpaired t-test, *p* < 0.001). The comparison of these experiments with those in which AITC was co-applied with A-967079 showed that the process was partially reversible. Quantification of the wound closure area revealed an average of 37.85 ± 4.17%, which was not significant compared to control conditions or to AITC-treated cells (n = 3, *p* > 0.05). To a lesser degree, cell migration diminished after 24 h of treatment with AITC from 71.49 ± 2.16% in the controls to 66.49 ± 1.48% (n = 6 experiments, Student’s unpaired t-test, p < 0.05). Addition of AITC and A-967079 in the medium determined total recovery of the migration rate compared to control, with a wound closure of 76.65 ± 3.2%, slightly higher than that of the untreated cells (n = 3, *p* < 0.05). Interestingly, treating the cells with the antagonist alone led to a small but significant increase in cell migration to 47.67 ± 1.26% after 12 h and even a higher increase, to 80.54 ± 0.77%, after 24 h. The augmentation of Panc-1 cells migration by approximately 15% from the control conditions indicates the presence of a constitutive basal activity in TRPA1 channels, which inhibited migration even under control conditions. As previously with microfluorimetry, a pool of 10% constitutively active channels may be responsible for this response. Moreover, these channels sparse and short bursts of spontaneous activity can add up during the 24 h measurement to have a significant effect on cell migration.

Previous studies showed that AITC diminished the viability and inhibited the migration of tumour cells through a mechanism that was not related to TRPA1 activation^16,17^. To determine whether similar behaviour occurred for Panc-1 cells, we used a MTT assay to determine cellular viability after 24 h of exposure to all compounds used in this study (Fig. 6C). Because these substances did not have an observed effect on cellular viability, we assume that the results of the experiments in which we use longer exposure times are related to activation or inhibition of TRPA1.

**Figure 7:**
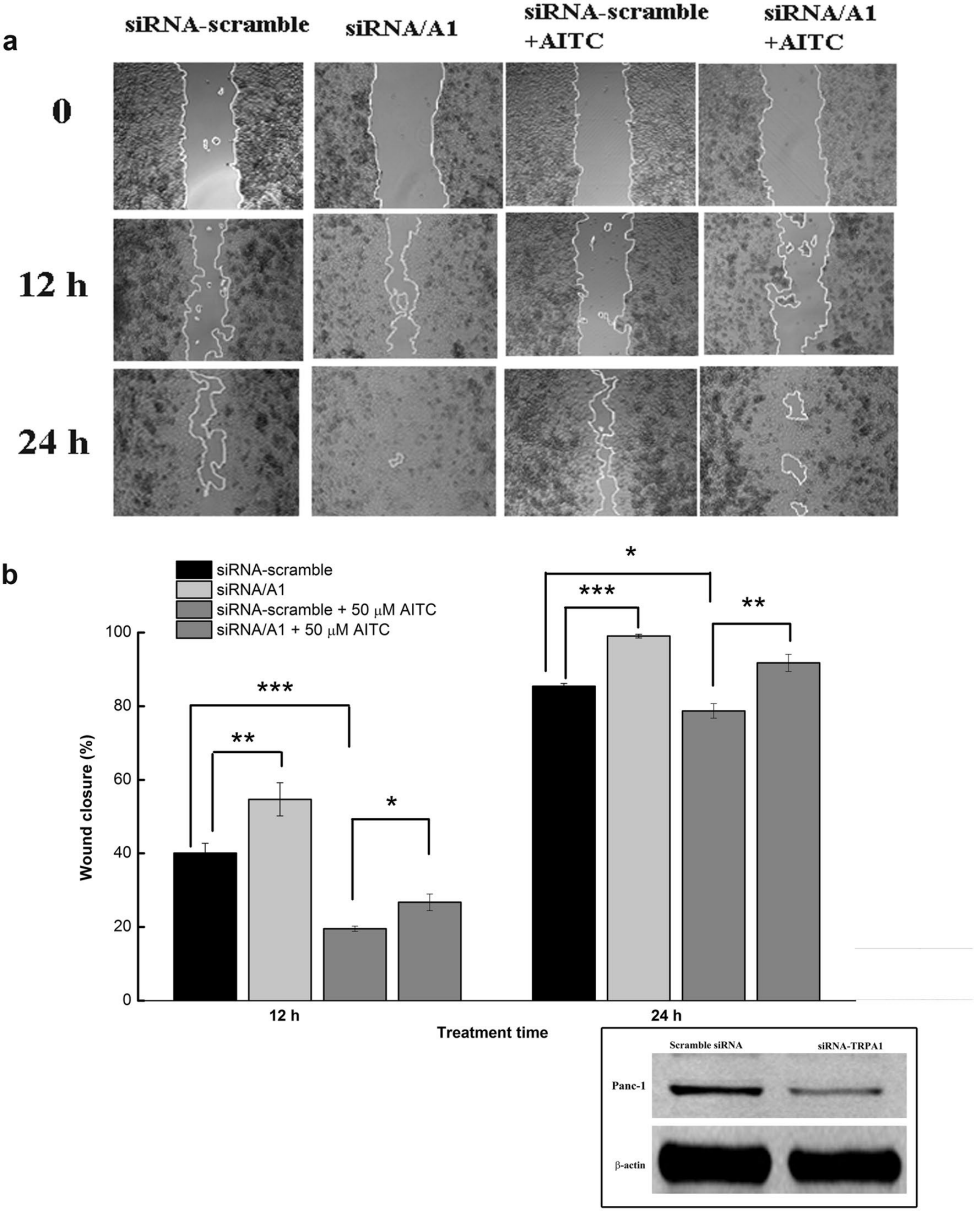
Effects of siRNA-mediated TRPA1 knockdown on the pharmacological sensitivity of Panc-1 cells. (**A**) Wound healing assay illustrating changes in the migration rates of Panc-1 cells 72 h after transfection with siRNA with and without AITC treatment. (**B**) Average ± SEM values from the three experiments performed in triplicate presented in (**A**). ns, not statistically significant; *p < 0.05; **p < 0.01; ***p < 0.001. Inset: Western blot showing silencing of TRPA1 in lysates collected at the end of the experiment.

To explore the effect of downregulating TRPA1 expression on Panc-1 cell migration, we used targeted siRNA experiments. The silencing efficiency (~ 58.7%) is shown in Fig. 7 (inset). Silencing TRPA1 expression induced a significant increase in the migration potential, from 40.08 ± 2.64% in cells transfected with scrambled RNA to 54.69 ± 7.54% in cells with silenced TRPA1 (n = 3, Student’s unpaired t-test, *p* < 0.01; Fig. 7). When we further analysed the capacity of AITC to block Panc-1 cell migration by comparing the effect of the agonist in control-scramble vs. siRNA/A1, we observed a significant decreases of 50.36 ± 5.39% in the migration efficiency after 12 h of treatment (Fig. 7, n = 3, Student’s unpaired t-test, *p* < 0.05) and of 7.35 ± 1.61% (Fig. 5, n = 3, Student’s unpaired t-test, *p* < 0.05) after 24 h of treatment (Fig. 7B).

Interestingly, silencing of endogenous TRPA1 was sufficient to promote increase of the migration rate, which supports our hypothesis that channels participate to migration using pore-independent signalling pathways, that remain to be investigated.

Another finding from this experiment is that the effect of AITC is higher under the control siRNA experimental (Fig. 7B) conditions than in cells that were not subjected to treatments with Lipofectamine 2000 and scrambled siRNA (as in Fig. 7A), suggesting that the transfection treatments could increase by themselves the TRPA1 constitutive basal activity. A more insightful way to determine whether TRPA1 indeed influences the tumorigenic process is to analyse the cell cycle progression because TRPA1 may mediate the calcium signals that impact this process, highlighting its role in carcinogenesis.

**Figure 8:**
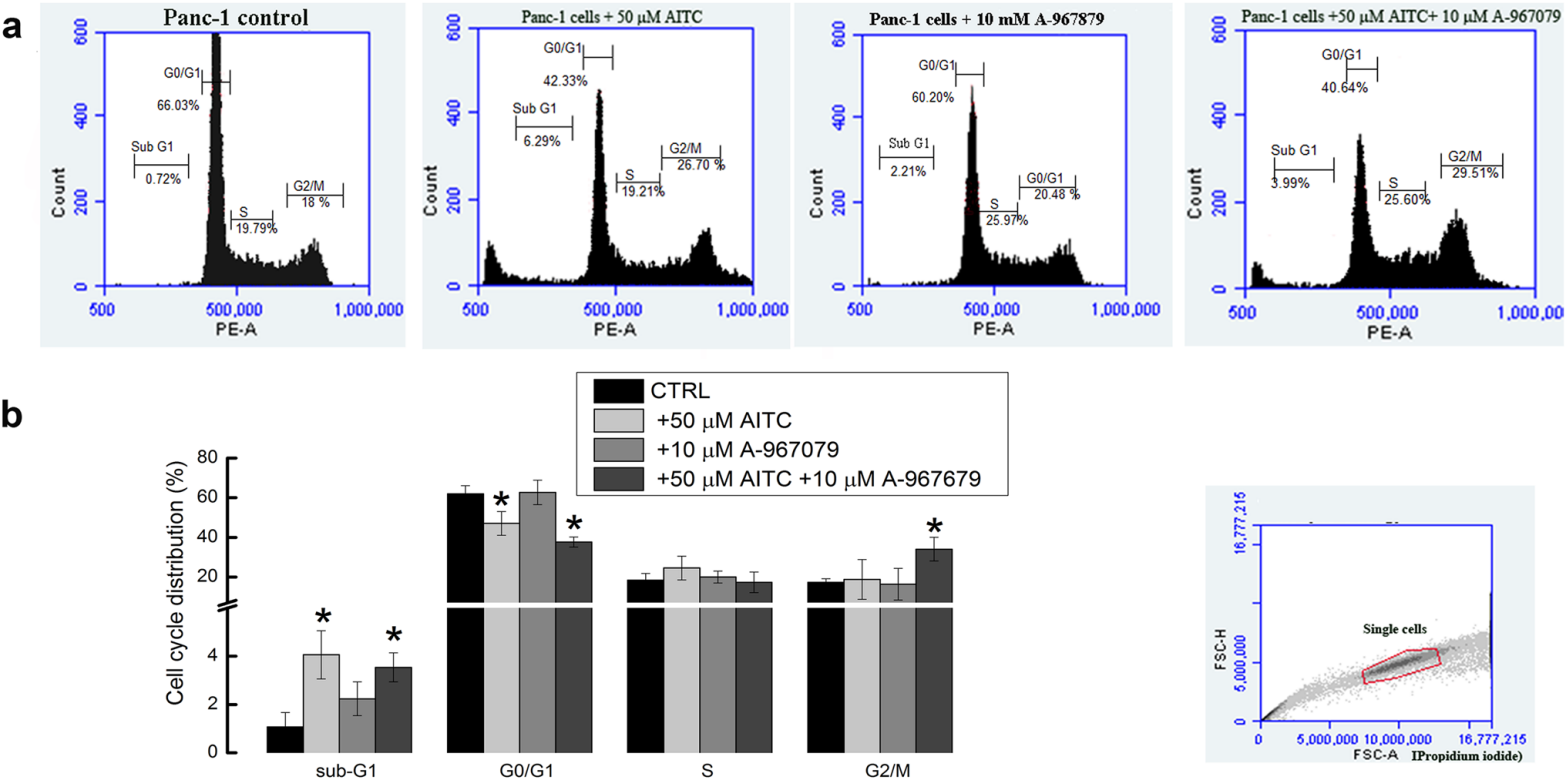
Influence of TRPA1 activation on cell cycle progression of Panc-1 cells. (**A**) Cell cycle distribution of Panc-1 cells under different conditions. Cells were cultured in complete medium and treated for 24 h with AITC (50 µM) or a mixture of AITC and A-967079 (10 µM), as indicated. (**B**) Summary of experiments presented in (**A**), showing the averages ± SEM of n = 3 experiments. Broken Y axes indicate numerically small, but significant differences of sub-G1 phase. The gating strategy of the control sample is presented in the inset.

We used flow cytometry to quantify the fraction of cells in the G0/G1, S and G2/M phases as a function of TRPA1 activation with 50 µM AITC, subsequent inhibition with 10 µM A-967079, and recovery with the mixture of both compounds as in the previous experiments. Measurements of nuclear staining with propidium iodide were used to estimate the number of cells in each phase as well as the number of cells in the sub-G1 phase (Fig. 8A). Quantification of the data showed that the application of 50 µM AITC to Panc-1 cells significantly (n = 3, Student’s unpaired t-test, p < 0.01) reduced their distribution in the G0/G1 phase from 61.94 ± 4.1% cells under control conditions to 47.07 ± 6% upon TRPA1 activation (Fig. 8B). A corresponding increase in the percentage of sub-G1 phase from 1.07 ± 0.6% of control cells to 4.83 ± 0.1% of AITC-treated cells was observed (n = 3, Student’s unpaired t-test, *p* < 0.05), with a slight but nonsignificant shift to the S phase. Application of 10 µm A-967079 did not change the cell distribution in any phase. Co-application of AITC and A-967079 for 24 h did not restore the initial conditions by sustaining the percentage of cells shifted from G0/G1 to sub-G1 phase to 3.54 ± 0.6%. An analysis of the data showed a slight but significant increase compared to the control cell population in the G2/M phase.

## Discussion

Numerous recent reports describe the involvement of TRP channels in the migration or proliferation of cancer cells, and although the results are conflicting depending on the tumour type studied, they support the putative role of TRP channels as therapeutic targets for cancer. Other reports insist on the role of some TRP proteins in the signalling pathways, through pore -independent contribution to the tumorigenic process^12^. This is an appealing hypothesis for TRPA1, which is a promiscuous channel activated by noxious, inflammatory or hypoxia, while the Src-dependent activation of ERK1/2 is well described^18^.

The study presented here demonstrates the presence of TRPA1 channels in PDAC cells and in normal pancreatic ductal cells. However, the expression level is different in PDAC cell lines, which indicate that the channel expression depends on intrinsic features of each cell line. For instance, MIA Paca-2 and Panc-1 relevant as in vitro models for PDAC, are both poorly differentiated cell lines^19^ but differ in tumorigenity, miRNA profile^20^, or expression of stem cell markers^21^. Likely others, yet unknown features also influence the expression of membrane proteins and particularly, of TRPA1.

The functionality of TRPA1 in Panc-1 subpopulation was demonstrated by the robust responses to the selective agonist AITC in both the calcium microfluorimetry and patch-clamp experiments. Co-expression with TRPM8 was investigated using the selective TRPM8 agonist WS-12^22^ and revealed little overlap in the cells responding to the two agonists, with TRPA1 having a more wide functional expression than TRPM8 (by approximately 3.7 times more, Fig. 2A). Ca^2+^ influx was the sole source of the intracellular Ca^2+^ increase following exposure to AITC (Fig. 3C). As shown in Fig. 4A, TRPA1 currents were observed at both positive and negative test potentials upon treatment with the specific agonist AITC, and the kinetics were dependent on extracellular calcium as described previously^23^. These results demonstrate that TRPA1 channels in Panc-1 cells share the same electrophysiological characteristics (a reversal potential of almost zero and outward rectification) and pharmacological properties (sensitivity to AITC and A-967079) as previously established for human TRPA1^24^.

Despite the robust currents upon activation, only a modest proportion of cells (27%) responded to the specific TRPA1 agonist in calcium microfluorimetry experiments. This behaviour contradicts immunofluorescence results, which show that about 90% of Panc-1 cells have a high TRPA1 expression. Apparently conflicting, these results indicate other factors to be crucial for channel functionality, such as the presence of endogenous constitutive channels, defects in trafficking or contribution to the tumoral process via channel-independent pathways. While endogenous constitutive activity of TRPA1 in Panc-1 cannot be excluded, in our hands this was minimal and exhibited by a small percentage of cells (Supplementary Fig. 2). It can be nevertheless imagined that during a 24 h wound healing assay, such sparse and short bursts of spontaneous activity can add up to have a significant effect on cell migration. Other various signals that regulate translocation to the membrane^25^ might represent one of the mechanisms controlling the TRPA1 function in tumoral cells.

However, a more valid assumption is that most of the TRPA1 channels that participate in the regulation of migration act through channel-independent mechanisms. Indeed, siRNA mediated-inhibition, activated migration of Panc-1 cells, as demonstrated in the wound healing assays. However, TRPA1 activation by AITC inhibited even more strongly the migration of Panc-1 cells, while application of the channel antagonist recovered the wound healing ability after 12 and 24 h. Taken together, these results validate the point that TRPA1 inhibits Panc-1 cells migration via channel dependent and independent pathways, consistent with previous reports^14,26^. The results of silencing experiments, which reveal that cell migration is inhibited with AITC, support the previously demonstrated antitumoral effects of the compound^16,17,27^. However, in our experiments, AITC did not inhibit cellular viability, which implies that its effect is more related to the TRPA1 activation, as siRNA knockdown is not 100% efficient.

With these results, it is difficult to conceive that, TRPA1 might be a reliable therapeutic target. However, PDAC is an extremely invasive cancer with a poor prognosis and resistant to therapies. As such, inhibiting cell migration by activation of TRPA1 might be a promising molecular tool for further investigations.

The relevance of TRPA1 in the tumorigenic process may be related to putative changes in the cell cycle progression, which is a hallmark of cancer. Following 24 h of incubation, AITC induced a redistribution of the cell cycle, resulting in a significant decrease in the G1/G0 population and a concomitant increase in the sub-G1 subpopulation. Identification of the sub-G1 phase may be used as an indicator of apoptotic cells in the cell cycle analysis^28^. With this hypothesis, the data correlate with previous results showing the apoptotic effects of AITC, but contradict the data showing the arrest in the M phase, which were presented in the same study^29^. We speculate that the G0-G1/S transition is limiting, and cells accumulate at this checkpoint, whereas activation of TRPA1 accelerates this transition. Alternatively, TRPA1 activation causes other cell cycle checkpoints to be more limiting, and therefore the overall cycle profile changes.

In conclusion, our study is the first to demonstrate that TRPA1 is expressed and functional in some PDAC cell lines. The channel activity is modulated by known agonists and antagonist compounds. Moreover, expression of this channel, both in a basal conduction state and when stimulated with the selective agonist AITC, inhibits cell migration through unelucidated signalling processes that we are currently investigating. The activated channel controls cell cycle progression and induces a sustained shift from G0/G1 to a sub-G1 phase.

Taken together, our results demonstrate that TRPA1 channels in the membrane of PDAC cells modulate the cellular processes by pore dependent and independent mechanisms. The putative role of the endogenous channels and activators open new perspectives for the study of TRPA1 in PDAC cells and in non-tumoral cell lines, which is currently under investigation in our group. With this new information we can anticipate the TRPA1 channel as a promising target for antitumor therapies of PDAC, along with other TRP family members.

## Methods

### Cell culture and transfection

Panc-1, Bx-PC3 and MIA Paca 2 cells were cultured in Dulbecco’s modified Eagle’s medium (DMEM) supplemented with 10% foetal bovine serum (FBS), GlutaMAX™ Supplement (Thermo Fisher Scientific), and 1% penicillin/streptomycin. The immortalized normal human pancreatic duct epithelial cells(HPDE) were cultured in keratinocyte serum-free medium supplemented with 0.2 ng/mL human recombinant epidermal growth factor and 30 µg/mL bovine pituitary extract (all from Thermo Fisher scientific) at the time of use. HEK-293 T cells were maintained in a 1:1 mixture of DMEM and Ham’s F12 medium (D8900, Sigma-Aldrich) supplemented with 10% FBS, 1% penicillin/streptomycin, and 1% L‐glutamine (all from Sigma-Aldrich). All cell cultures were maintained at 37 °C in a humidified atmosphere of 5% CO_2_ in air.

Recombinant human TRPA1 DNA (5 μg/25 cm^2^ flask) was transiently transfected into HEK-293 T cells using jetPEI (8.5 μL) transfection reagent (Polyplus transfection, Illkirch, France) when cells were 60–70% confluent. pcDNA3.1 plasmid containing the human TRPA1 sequence was a kind gift from Dr. Gordon Reid, University College Cork.

### Immunocytochemistry

Cells were plated at 10^4^ cells/ml on poly-D-lysine coated glass cover slips in DMEM growth medium without antibiotic and allowed to attach overnight at 37 °C. The samples were then fixed with 4% paraformaldehyde for 20 min at room temperature (RT) and subsequently incubated for 3 min with 0.2% Triton-X-100 to induce cell membrane permeabilization. For immunostaining, the cells were preincubated with 0.5% BSA blocking solution for 45 min, and then incubated for 1 h at 4 °C in mouse monoclonal TRPA1 primary antibody (sc-376495, Santa Cruz Biotechnology) diluted 1:200 in BSA, and subsequently with corresponding AlexaFluor conjugated secondary antibodies (A11034, Invitrogen, diluted 1:1000 in PBS) for 30 min. Samples were washed three times (5 min each) in cold HBSS prior each step of the imunofluorescence protocol. Nuclei were stained with DAPI for 15 min and coverglass were then mounted onto slides using FluoSave reagent. Mounted slides were kept overnight at 4 °C, in the dark, before image acquisition. Fluorescence and DIC images were acquired using a Zeiss Axio Imager Z1 with ApoTome Module (Berlin, Germany), using a 63 × objective with oil. The ImageJ software suite was used for background subtraction, scaling the fluorescence intensity, and merging the color channels.

### Viability assay MTT based

Panc-1 cells used for screening of the effect of chemical agents on cell viability, were seeded at 10^4^ cells/ml on 96 well plate and maintained overnight in DMEM growth medium without antibiotic. Cells were then treated for 24 h with 50 µM AITC or 10 µM A967079, respectively a mixed solution between the two compounds. Each test has also included a blank containing complete medium without cells and a negative control represented by untreated cells. Subsequently, cells were incubated in culture medium containing 10% MTT (TOX-1KT, Sigma) reconstituted in DMEM. Formazan crystals formed after 4 h incubation at 37 °C were dissolved by adding an amount of MTT Solubilization Solution (M-8910, Sigma) equal to the original culture medium volume (100 µL/well) and gentle mixing in a gyratory shaker for 20 min in the dark. Absorbance was spectrophotometrically measured at a wavelength of 570 nm. The background absorbance of multiwell plates was read at 690 nm. The mean blank absorbance and background absorbance were subtracted from the 570 nm measurement. The results were tested for a statistically significant difference between using Student’s t Test and graphs were generated in OriginPro8 program.

### Calcium microfluorimetry

Panc-1 cells were plated on Poly-D-lysine-coated glass round coverslips (25 mm diameter) and incubated in standard extracellular solution (see the solution composition below) containing 2 µM Calcium Green-1 AM and 0.02% Pluronic F-127 (both from Thermo Fisher Scientific) for 30 min at 37 °C and allowed to recover for another 30 min before use. The coverslips were mounted in a Teflon chamber (MSC TD, Digitimer, Welwyn Garden City, UK) on the stage of an Olympus IX70 inverted microscope. Cells were illuminated at 470 nm with a Dual OptoLED light source (Cairn Research, Faversham, UK) controlled by Axon Imaging Workbench 2.2 software (Axon Instruments, Union City, CA, USA), which was also used for image acquisition and analysis. Fluorescence image sequences were acquired with a CCD camera (Cohu 4910, Pieper GmbH, Schwerte, Germany) and digitized to 8 bits. The changes in fluorescence were analysed with custom software. The maximal fluorescence change during stimulation relative to the initial fluorescence intensity (ΔF/F_0_) was computed by measuring individual F_0_ values before each stimulus. The ΔF/F_0_ values were also called "amplitudes". The standard extracellular solution contained the following (in mM): NaCl, 140; KCl, 4; MgCl_2_, 1; CaCl_2_, 2; HEPES, 10; NaOH, 4.54; and glucose, 5 (pH 7.4 at 25 °C). The calcium-free extracellular solution contained the following (in mM): NaCl, 140; KCl, 4; MgCl_2_, 1; EGTA, 1; HEPES, 10 (pH adjusted to 7.4 (at 25 °C) with NaOH). The pharmacological compounds were added from stock solutions. AITC (100 mM stock in DMSO) was obtained from Fluka, and A-967079 (100 mM stock in DMSO) was obtained from Tocris Bioscience.

### Electrophysiology

For electrophysiological analysis, cells were cultured in accordance with the same protocol used for calcium microfluorimetry. Whole-cell patch-clamp recordings in voltage clamp mode were performed using a WPC-100 patch clamp amplifier (E.S.F. Electronic, Göttingen, Germany). Borosilicate capillaries with filaments (BF150-86-10HP, Sutter Instrument, Novato, CA, USA) were pulled using a pipette puller (P-1000, Sutter Instrument), and the pipette tips were polished to provide a resistance of 2–4 MΩ. The composition of the calcium-containing extracellular solution was the same as that used in the calcium microfluorimetry experiments. The calcium-free extracellular solution contained the following (in mM): NaCl, 140; KCl, 4; MgCl_2_, 1; EGTA, 1; HEPES, 10 (pH-adjusted to 7.4 at 25 °C with NaOH). The intracellular solution contained the following (in mM): K^+^ gluconate, 134; MgCl_2_, 1; EGTA, 5; Na_2_ATP_3_, 2; NaCl, 6; HEPES, 10 (pH-adjusted to 7.2 at 25 °C with KOH). The bath electrode was placed in the same intracellular solution above an agar bridge of extracellular solution to ensure symmetrical junction potentials at the two electrodes. Capacitive transients were compensated using the R-series, C-slow and C-fast adjustments of the amplifier until the 20 mV square test pulse could be reproduced. The signal was filtered at 3 kHz and digitized at 5 kHz using an Axon Instruments Digi Data 1322A interface controlled by pCLAMP 8.2 (Molecular Devices, Sunnyvale, CA, USA). For recordings in calcium-containing solution, the voltage clamp protocols consisted of ramps from − 80 mV to 80 mV (from − 80 mV, 200 ms every 5 s), while ramps from − 100 mV to 100 mV (from − 80 mV, 400 ms every 2 s) were used for recordings in calcium-free solution.

### RNA interference-mediated gene silencing

Panc-1 cells were grown to approximately 70% confluence in antibiotic-free medium and then transfected with siRNA—Lipofectamine 2000 (11668027, Antisel) pre-mixed complex containing 60 nM siRNA directed against human TRPA1or Scrambled siRNA (Thermo Fisher Scientific) according to the manufacturer’s instructions. The transfection complexes were prepared and delivered in Opti-MEM (cat. no. 31985062, Gibco, Antisel) reduced-serum medium and replenished with 10% serum-containing media after 8 h to allow cell recover. Further, a second transfection was performed in the same conditions. To verify TRPA1 knockdown, the total protein fraction was extracted after 72 h of transfection and analysed using Western blotting. The specific siRNA sequence targeting TRPA1 (siRNA/A1) was 5′-GGAUGUUAUAUAUGAACCGtt (cat. no. AM16708, Ambion). As negative control (siScramble) was used a scrambled siRNA sequence (Ambion, AM4636).

### Western blotting

Cells were lysed for 30 min on ice in 1% Triton-X100 containing buffer (1,5 mM MgCl_2_, 150 mM NaCl, 1 mM EDTA and 5 mM HEPES pH 7,4) supplemented with 1 × protease inhibitor cocktail from Roche. Cell lysates were cleared by centrifugation for 20 min at 14,000 × g and 4 °C. Equal amounts of proteins (50 µg) from each sample assessed by bicinchoninic acid (BCA) were separated by SDS-PAGE in 8% polyacrylamide gels and transferred onto nitrocellulose membranes. The membranes were blocked for 1 h at room temperature in 10% nonfat dry milk and then probed with primary rabbit polyclonal TRPA1 (PA1-46159, Thermo Fisher Scientific) antibody, which was diluted (1:1000) in 5% milk dissolved in phosphate buffered saline (PBS), and incubated overnight at 4 °C. The protein was then detected with goat anti-rabbit (sc-2004) coupled with horseradish peroxidase enzyme (HRP) and diluted (1:10,000) in the same buffer as the primary antibody for 1 h at RT. Subsequently, membranes were soaked in mouse anti-tubulin (1:1000, T6199_Sigma) or anti-HSP 90α/β (sc-13119, Santa Cruz Biotechnology) antibodies as the loading control. The results were viewed by chemiluminescent reaction using the Pierce ECL substrate (32106, Thermo Fisher Scientific). Band intensity was quantifed using NIH ImageJ software and the results were then normalised to the non-tumoral HPDE cells.

### Wound healing assay

Cells were grown to more than 90% confluence to form a monolayer in a 6-well plate at 37 °C. A linear wound was created with a sterile pipette tip of 200 µM in the centre of the wells. The wells were washed twice with PBS, to remove floating cells, and medium containing test compounds (50 µM AITC, 10 µM A967079) was then added. Cells were monitored using phase contrast at 4 × objective magnification. The area covered by cells that migrated into the scratch within 12 and 24 h was measured using the ImageJ software, and every cell that moved towards the wound was considered. Up to 20 fields (entire wound) per well were selected for analysis. Each condition was set up in triplicate, and the experiments were performed three times (n = 3).

### Quantitative PCR (qPCR)

Total RNA from HPDE, Panc-1, MIA PaCa-2, HEK/A1, BxPC-3 and HEK/WT cell lines were isolated with Trizol reagent (Invitrogen, USA) according to the manufacturer’s instructions. A reverse transcription PCR (rtPCR) was performed with High Capacity cDNA Reverse Transcription Kit (Applied Biosystems, USA). qPCR amplification was performed in triplicate for each sample with TaqMan Universal PCR Master Mix (Applied Biosystems, USA) in a total volume of 25 μland cDNA concentration of 5 ng/µl. The level of each TRPA1 mRNA (ref Hs00175798_m1, Dye: FAM-MGB, ThermoFisher Scientific) was normalized to endogenous control Hu18S (ref Hs99999901_s1, Dye: FAM-MGB, ThermoFisher Scientific) and fold changes were determined within tumoral cells compared with paired HPDE non-tumoral cells. Data were analysed with SDS 1.4 software using comparative Ct method [2^(-delta delta Ct)].

### Cell cycle analysis

Panc-1 cells were seeded in Petri dishes at a density of 10^6^ cells and incubated in a 5% CO_2_ incubator at 37 °C. Subconfluent cells were treated for 24 h with AITC and A-967079 in culture medium as described above. All the treatments were performed in three replicates. Cells heat-treated for 1 h at 56 °C were used as positive controls, and untreated viable cells were used as negative controls. Cells were then harvested, washed with cold PBS (without calcium chloride and magnesium chloride) and processed for cell cycle analysis. Briefly, 10^6^ cells were resuspended in 200 µL of cold PBS supplemented with 2% FBS, and then 600 µL of cold ethanol was added. The cells were then incubated at − 20 °C for at least 48 h. After centrifugation at 1500 rpm, the pellet was washed three times, resuspended in 200 µL of PBS, stained with 50 µg/mL propidium iodide, treated with 10 µg/mL RNase, and incubated for 1 h in the dark at 37 °C. Then, all the samples were analysed using the Accuri C6 Plus instrument (Becton, Dickinson and Company, San Jose, CA, USA) equipped with two lasers emitting at 488 and 635 nm. The instrument was controlled before acquisition using the standard CS&T‐bead protocol (BD™ CS&T RUO Beads). A total of 10,000 single cells for each sample were acquired at a low flow rate, and the propidium iodide fluorescence was detected through a 585/40-nm bandpass filter.The histogram of DNA content was obtained by gating the cell population on a fluorescence intensity versus forward scatter dot plot. Thereafter, analysis of the cell cycle distributions was performed using BD Accuri C6 Plus software (Becton, Dickinson & Company).

### Statistical analysis

Data are expressed as the mean ± SEM. A paired Student’s t-test was used to compare the relative migration area in treated and non-treated cells, as well as in siRNA-scramble vs siRNA/A1 experiments. A value of *p* < 0.05 was considered significant.

## Supplementary Information

Supplementary Figure S1.

Supplementary Figure S2.
