# Publisher Correction: Functional expression of the transient receptor potential ankyrin type 1 channel in pancreatic adenocarcinoma cells

**DOI:** 10.1038/s41598-021-88169-9

**Published:** 2021-04-19

**Authors:** Florentina Cojocaru, Tudor Şelescu, Dan Domocoş, Luminiţa Măruţescu, Gabriela Chiritoiu, Nicoleta-Raluca Chelaru, Simona Dima, Dan Mihăilescu, Alexandru Babes, Dana Cucu

**Affiliations:** 1 Department DAFAB, Faculty of Biology, University of Bucharest, Splaiul Independenței 91-95, Bucharest, Romania; 2 Faculty of Biology, Research Institute of the University of Bucharest (ICUB), University of Bucharest, Bucharest, Romania; 3 Department of Molecular Cell Biology, Institute of Biochemistry, Romanian Academy, Splaiul Independenței 296, 060031 Bucharest, Romania; 4 Center of Excellence in Translational Medicine, Fundeni Clinical Institute, 022328 Bucharest, Romania

Correction to: *Scientific Reports* https://doi.org/10.1038/s41598-021-81250-3, published online 21 January 2021

In the original version of this Article Florentina Cojocaru and Tudor Șelescu were omitted as equally contributing authors. In addition, the Author Contributions section in this Article was incorrect.

“F.C. performed the cell cultures, migration experiments, siRNA and data analysis. G.C performed Western blotting. T.S. performed patch-clamp and data analysis. D.D. performed the microfluorimetry experiments. D.M. and A.B. analysed and interpreted the data. L.M. performed the cell cycle experiments. R.C performed qRT-PCR experiments. S.D provided PDAC and HPDE cell lines ann interpreted qRT-PCR experiments. F.C., T.S., D.C. and A.B. analysed the data and wrote the manuscript. All authors read and approved the final version of the manuscript.”

now reads:

"Florentina Cojocaru and Tudor Selescu contributed equally and are considered first authors in this publication. F.C. performed the cell cultures, migration experiments, siRNA and data analysis. T.S. performed microfluorimetry experiments, patch-clamp recordings and data analysis. D.D. performed microfluorimetry experiments. L.M. performed the cell cycle experiments. G.C. performed Western blotting. N.R.C. performed qRT-PCR experiments. S.D. provided the PDAC and HPDE cell lines and interpreted qRT-PCR experiments. D.M. and A.B. analyzed and interpreted the data. F.C., T.S., D.C. and A.B. analyzed the data and wrote the manuscript. All authors read and approved the final version of the manuscript."

The original Article has been corrected.

