# Supplementary material for: Functional expression of the transient receptor potential ankyrin type 1 channel in pancreatic adenocarcinoma cells
Source: Sci Rep. 2021 Jan 21;11:2018. doi: 10.1038/s41598-021-81250-3 (PMC7819973; doi:10.1038/s41598-021-81250-3)
Supplement: Supplementary file 1 — Supplementary Figure S1. [file 41598_2021_81250_MOESM1_ESM.pdf]

# Functional expression of the transient receptor potential ankyrin type 1 channel in pancreatic adenocarcinoma cells

Florentina Cojocaru[1*], Tudor Șelescu[1*], Dan Domocoş[1], Luminiţa Măruţescu[2], Gabriela Chiritoiu[3], Nicoleta-Raluca Chelaru[4], Simona Dima[4], Dan Mihăilescu[1], Alexandru Babes[1✉], Dana Cucu[1✉]

1. Department DAFAB, Faculty of Biology, University of Bucharest, Splaiul Independenței 91-95, Bucharest Romania

2. Faculty of Biology, Research Institute of the University of Bucharest (ICUB), University of Bucharest, Bucharest, Romania

3. Department of Molecular Cell Biology, Institute of Biochemistry, Romanian Academy, Splaiul Independenței 296, 060031 Bucharest, Romania

4. Center of Excellence in Translational Medicine, Fundeni Clinical Institute, 022328 Bucharest, Romania

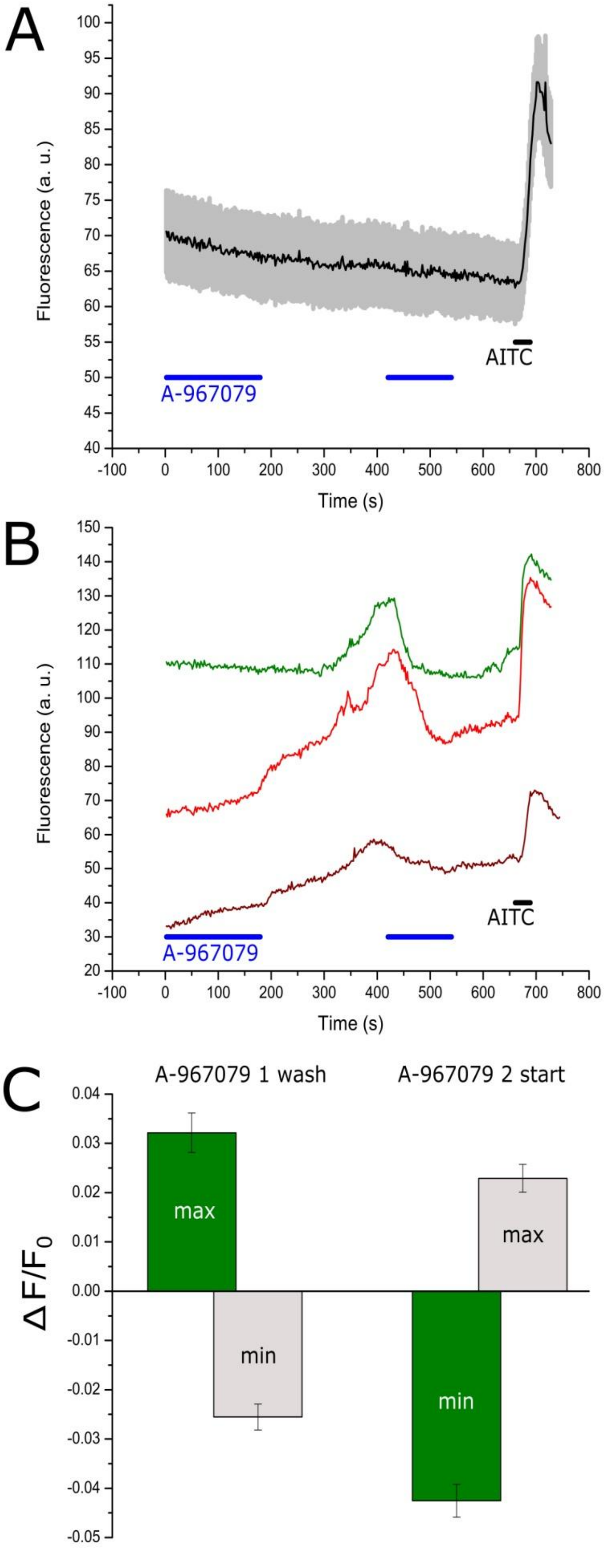
A
B
C
Fluorescence (a. u.)
Time (s)
A-967079
AITC
ΔF/F0
A-967079 1 wash
A-967079 2 start
max
min
max
min

**Fig. S2 Testing TRPA1 constitutive activity in Panc-1 cells using the A-967079 antagonist and calcium imaging**.

A. The recordings started with the cells superfused with A-967079 (10 µM ) for 3 minutes, continued with a 4 minute wash-out segment, after which A-967079 was applied a second time, for 2 minutes. AITC (100 µM, 30 s) was applied at the end. The trace shows the average fluorescence ± SEM of 34 AITC-sensitive cells (background corrected).

B. Only 7 from the 78 AITC-sensitive cells analyzed responded with $\Delta F/F_0$ amplitudes larger than ± 10% to A-967079 wash-out and/or start. Three exemplary traces are displayed, showing small increases in fluorescence upon antagonist wash-out, spontaneous activity during antagonist's absence and a partial inhibition of this activity by the second A-967079 application.

C. The $\Delta F/F_0$ amplitudes for the maximal increase (max) and maximal decrease (min) in fluorescence during the first minute of A-967079 wash-out and the first minute from the start of the second A-967079 challenge. The $\Delta F/F_0$ values for the antagonist wash-out were 3.2 ± 0.4% maximal increase and 2.5 ± 0.3% maximal decrease. The $\Delta F/F_0$ values for the second antagonist application were 4.2 ± 0.3% maximal decrease and 2.3 ± 0.3% maximal increase (averages ± SEM, n=78 AITC-sensitive cells).
