# Supplementary material for: Functional expression of the transient receptor potential ankyrin type 1 channel in pancreatic adenocarcinoma cells
Source: Sci Rep. 2021 Jan 21;11:2018. doi: 10.1038/s41598-021-81250-3 (PMC7819973; doi:10.1038/s41598-021-81250-3)
Supplement: Supplementary file 2 — Supplementary Figure S2. [file 41598_2021_81250_MOESM2_ESM.pdf]

Functional expression of the transient receptor potential ankyrin type 1 channel in pancreatic adenocarcinoma cells

Florentina Cojocaru[1*], Tudor Șelescu[1*], Dan Domocoş[1], Luminiţa Măruţescu[2], Gabriela Chiritoiu[3], Nicoleta-Raluca Chelaru[4], Simona Dima[4], Dan Mihăilescu[1], Alexandru Babes[1✉], Dana Cucu[1✉]

1. Department DAFAB, Faculty of Biology, University of Bucharest, Splaiul Independenței 91-95, Bucharest Romania

2. Faculty of Biology, Research Institute of the University of Bucharest (ICUB), University of Bucharest, Bucharest, Romania

3. Department of Molecular Cell Biology, Institute of Biochemistry, Romanian Academy, Splaiul Independenței 296, 060031 Bucharest, Romania

4. Center of Excellence in Translational Medicine, Fundeni Clinical Institute, 022328 Bucharest, Romania

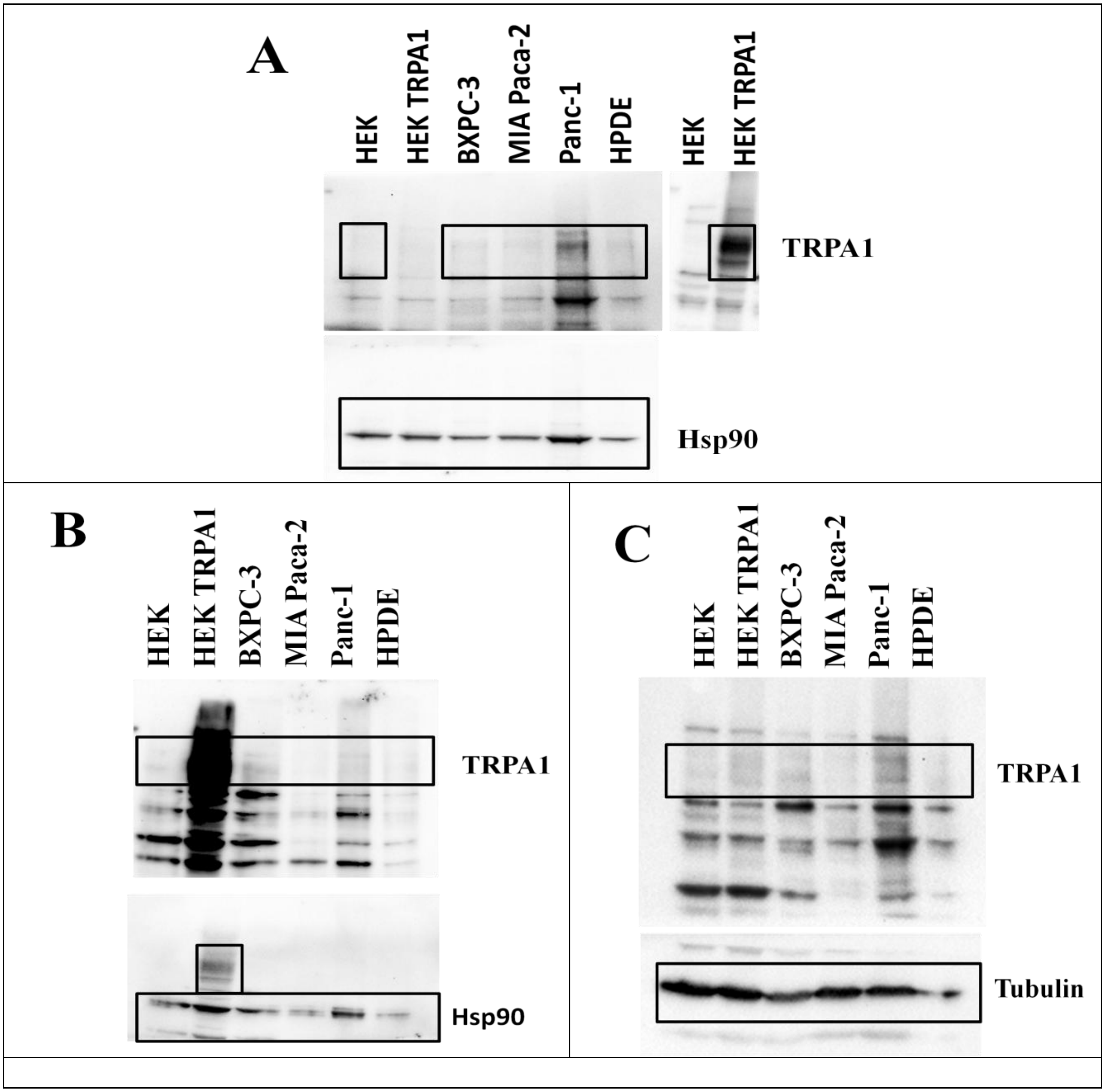
A
HEK
HEK TRPA1
BXPC-3
MIA Paca-2
Panc-1
HPDE
HEK
HEK TRPA1
TRPA1
Hsp90
B
HEK
HEK TRPA1
BXPC-3
MIA Paca-2
Panc-1
HPDE
TRPA1
Hsp90
C
HEK
HEK TRPA1
BXPC-3
MIA Paca-2
Panc-1
HPDE
TRPA1
Tubulin

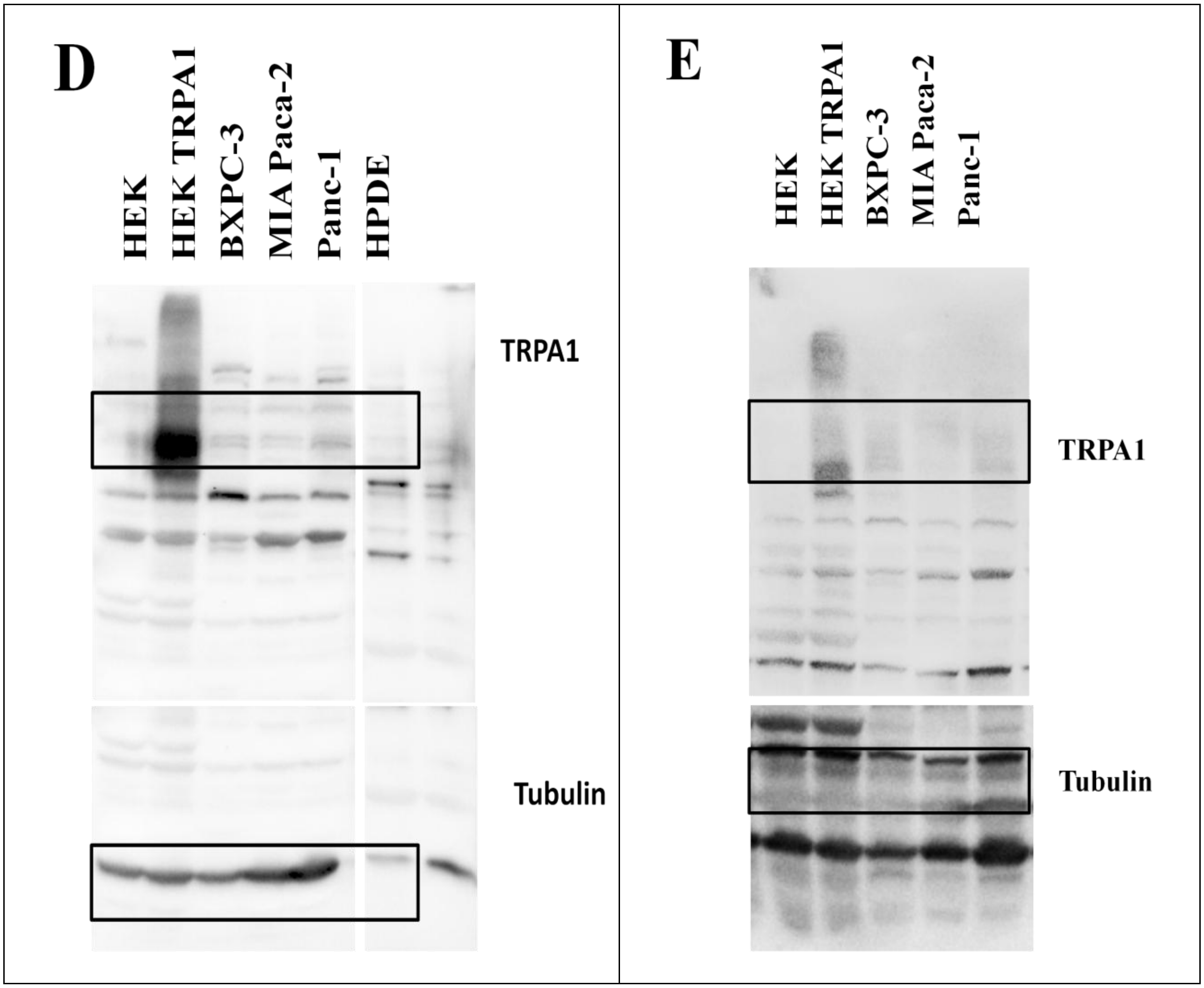

D
HEK
HEK TRPA1
BXPC-3
MIA Paca-2
Panc-1
HPDE
TRPA1
Tubulin
E
HEK
HEK TRPA1
BXPC-3
MIA Paca-2
Panc-1
TRPA1
Tubulin

Fig. S1 Western blotting evaluation of the expression of TRPA1 protein in pancreatic cancer cell lines.

A. Original data for the representative images presented in the manuscript. B-E. Quantifiable TRPA1 protein expression data. The highest protein levels was detected by western blot analysis in Panc-1 cells, the second highest levels in BxPC-3 cells and the lowest level was in MIA PaCa-2 cells. Tubulin or HSP90 was used for normalization of protein loading.
